# Does China’s Equalization of Basic Public Health Services policy improve delivery care for migrant women?

**DOI:** 10.1186/s12889-022-14950-8

**Published:** 2023-01-11

**Authors:** Hong Zou, Han Xiao, Hongwei Xu

**Affiliations:** 1 School of Economics, Southwestern University of Finance and Economics, 555 Liutai Ave, Wenjiang District, Chengdu, China; 2 Department of Sociology, Queens College, Powdermaker Hall 252, 65-30 Kissena Blvd, 11367 Queens, NY USA

**Keywords:** China, Delivery care, Migration, Woman

## Abstract

**Background:**

As of 2020, 1 in 4 people in China is a domestic migrant. However, their lack of access to health care in destination cities has been largely neglected by the Chinese government until recently.

**Methods:**

Drawing on data from the 2010–2016 China Migrants Dynamic Survey, this study evaluated the impact of a pilot program of the Equalization of Basic Public Health Services launched in 2014 and focused on place of childbirth, an important indicator of delivery care. A difference-in-differences design was employed for statistical inference.

**Results:**

The migrant pilot program increased the likelihood of a migrant childbirth at a migration destination by about 4 to 8 percentage points, depending on the model specification. Further analyses revealed that this positive effect was short-term and benefited relatively better-off migrant families.

**Conclusion:**

The migrant pilot program improved delivery care for migrant women. The Chinese government needs to expand this pilot program to more cities and improve its benefits to better serve the massive migrant population.

**Supplementary Information:**

The online version contains supplementary material available at 10.1186/s12889-022-14950-8.

## Background

China’s domestic migrant population has tripled from 121 million in 2000 to 376 million in 2020, accounting for more than a quarter of the country’s population [1]. The majority of these migrants—estimated to be 174 million as of 2019 [2]—are rural-to-urban migrant workers. Despite the large scale of domestic migration, the household registration system, known as *hukou*, has remained an institutional barrier to rural-to-urban migrant workers, restricting their equal access to job opportunities, pension, housing market, and public schools for their children [3, 4]. With respect to health care, the vast majority of rural-to-urban migrant workers are officially enrolled in the New Rural Cooperative Medical System, a health insurance system that operates in their rural home villages [5]. The lack of access to urban health insurance systems has severely deterred migrants from seeking health care in their destination cities [6]. As a result, it is not surprising that China’s rural-to-urban migrants are facing various health disadvantages compared to their urban counterparts [7–11]. In addition, case studies in Beijing and Guangzhou have documented legal, cultural, and language barriers for international immigrants to access affordable healthcare services [12, 13].

In response to deteriorated health equity, the Chinese government has introduced the Equalization of Basic Public Health Services (EBPHS) program as part of its comprehensive health system reform that was launched in 2009 [14]. One of the two main goals of the EBPHS is to provide universal coverage of basic public health services for all Chinese citizens. In practice, primary health care institutions, including community health centers and stations in urban areas and township hospitals and village clinics in rural areas, are responsible for delivering these services to local residents free of charge. The financial costs are shared between the central and local governments. The EBPHS initially targeted four categories of basic public health services: vaccination, infectious disease surveillance, young child (0–3 years old) health management, and maternal health management [14, 15]. However, similar to the New Rural Cooperative Medical System, implementation of the EBPHS was rooted in local residents’ hukou registration status. Hence, rural-to-urban migrants were forced to seek these basic health services covered by the EBPHS in their rural home communities rather than their urban migration destinations. Even urban-to-urban migrants have to resort to seeking care in their hometowns where their hukou is registered.

In December 2013, the former National Health and Family Planning Commission of China (now the National Health Commission) announced a pilot program to promote the EBPHS for all domestic migrants living in 40 selected cities (see Additional file 1: Table S1) across 27 provinces or their administrative equivalents (municipalities and autonomous regions) [16]. According to this pilot program, primary health care institutions in urban areas are responsible for providing the basic public health services specified in the EBPHS to all migrants based on their current place of residence, regardless of their rural–urban origin or place of hukou registration. Certain public health services were given top priority, including vaccination of migrant children, family planning, maternal and child health care, health education, establishing health records, and prevention and control of infectious diseases. The migrant pilot program officially rolled out in March 2014. In November 2014, the former National Health and Family Planning Commission of China issued a joint guideline with the Ministry of Civil Affairs, the Ministry of Finance, and the Office of Migrant Workers at the State Council to promote the EBPHS for all migrants [15]. This joint guideline called for coordination among different government agencies to implement the EBPHS for migrants and tasked urban community health care facilities to serve migrants living in local communities. This joint guideline also declared six targets of universal or near-universal coverage of the EBPHS for migrants by 2020: 95% vaccination rate for migrant children, 100% reporting rate of infectious diseases among migrants, established electronic medical records for 80% of migrants, 95% coverage rate of free consulting for family planning, and 100% coverage rate of free birth control service for migrant women.

To date, only a handful of studies have attempted to evaluate the performance of the EBPHS [15, 17–22]. However, most of these studies relied heavily on aggregated data at the provincial level and descriptive statistics to examine temporal trends or cross-sectional regional variations. Therefore, these studies did not provide any formal evaluation of the causal impact of the EBPHS. With respect to health indicators, prior research is often limited to child vaccination, establishing medical records for children and pregnant women, and management of hypertension and diabetes for older adults [19–21]. These indicators are measured from administrative data collected at health care facilities and do not capture people lacking access to health care.

Only one study has used microdata to examine the effects of the EBPHS on migrants’ healthcare utilization and health status [22]. Using the 2013 and 2014 waves of data from the China Migrants Dynamic Survey (CMDS), Fu et al. found that the EBPHS pilot program increased the probabilities for China’s domestic migrants to establish electronic health records and to reimburse their last inpatient visits, as well as reduced the probability of self-reported injury in the past year. However, they only examined a short period of two years during which the EBPHS pilot program was rolling out and did not focus on maternal and child healthcare services, two of the targeted public health areas of the program.

Combining data from a nationally representative survey of domestic migrants, statistical yearbooks, and several statistical databases, the current study is among the first to estimate the causal impact of the EBPHS program on maternal health. Given migrant women’s long-lasting disadvantage in accessing basic health services, the policy evaluation focused on their maternal health care and childbirth outcomes. Using the 40 cities designated by the EBPHS migrant pilot program to identify the treatment group, this study adopted a difference-in-differences (DID) strategy to formally evaluate the performance of the program.

Given that the EBPHS pilot program was announced in December 2013, and the selected cities started to roll out the pilot program in March 2014 after three months of preparations, we expect that:

- *Hypothesis 1: The EBPHS migrant pilot program had a positive effect on maternal health care in 2014 and later.*

According to government bulletins and news reports, several provinces (including Shandong, Liaoning, Jilin, Shanghai, Hunan, Hubei, and Fujian) began to expand the EBPHS migrant pilot program in the late 2015 and reached nearly universal coverage by the end of 2016.^1^ The expansion of the EBPHS program could narrow the gap in maternal health care between the pilot cities and those in our control group. Therefore, we expect that:

- *Hypothesis 2: The positive effect of the EBPHS migrant pilot program diminished over time.*

## Methods

### Data and sample

The primary data used in this study came from the 2010–2016 China Migrants Dynamic Survey (CMDS), a repeated cross-sectional survey conducted by the National Health Commission of China. Using a stratified, multistage probability proportional to size sampling strategy, the CMDS draws a nationally representative sample of about 130,000-200,000 domestic migrants who are Chinese citizens and aged between 15 and 59 years old annually from more than 1,500 cities and counties in 31 provinces (or their administrative equivalents) and Xinjiang Production and Construction Corps in mainland China [23]. In the CMDS sample, a domestic migrant is defined as someone who has lived in the current city or county for 1 month or longer at the time of interview but whose hukou is registered somewhere else. The pilot survey took place in 2009, and the national full-scale survey was launched in 2010 and repeated annually since then. This study drew on individual-level data from the 2010–2016 waves of the CMDS. The 2017 wave did not collect data on migrant women’s fertility history, making it impossible to identify the outcome variable in this study, and hence it was excluded. Data from more recent waves of the CMDS are not yet publicly available, preventing an investigation into the long-term effects of the EBPHS program.

Childbirth was used as the unit of analysis because each migrant woman could have more than one birth during the study period. To derive the analytic sample, this study first identified a nationwide sample of 49,881 migrant women who had 50,365 births in the year of the interview or the year before (i.e., between 2009—the year prior to the CMDS national baseline—and 2016). Among them, 21,966 births were delivered by 21,755 migrant women somewhere else rather than in any treatment or control city (see the Measures section), and hence, they were excluded from the analysis. The final analytic sample included 28,126 births by 28,399 migrant women in 40 treatment cities and 51 control cities.

### Measures

The outcome variable was place of delivery, an indicator of reproductive health care that may be a matter of life and death for pregnant migrant women and their unborn children [24–27]. This dichotomous variable was coded 1 if a birth was delivered by a migrant woman at her migration destination and 0 otherwise. In China, compared with migrant origins in rural areas or small towns, the quality of prenatal, delivery, and postnatal care is generally better at designation cities where childbirths are tightly regulated to take place at a hospital (rather than at home or a clinic) with modern medical equipment and skilled birth attendants who can better handle childbirth complications [28]. Chinese migrant mothers and their children are more likely to receive public health services such as postnatal home visits and free vaccinations [29]. As a result, maternal and infant mortality risks likely would be significantly higher had migrant women given birth in their hometowns [29, 30]. Residential mobility during pregnancy has also been associated with poor maternal, prenatal, and postnatal health outcomes [24, 31]. In fact, the negative health experiences and outcomes associated with return migration for childbirth was the main driving factor behind the 2013 EBPHS migrant pilot program and the 2014 joint guideline to promote migrant women’s equal access to reproductive health services as local permanent residents [24]. Therefore, place of delivery is an ideal indicator to assess the implementation of the EBPHS migrant pilot program.

The treatment in this study was the EBPHS migrant pilot program that was announced in November 2013 and rolled out in March 2014. Therefore, the time period prior to (and including) March 2014 was defined as pre-treatment, whereas the period after March 2014 was considered as post-treatment. The 40 cities that were chosen to pilot the EBPHS migrant program were the treatment cities, and the births given by migrant women in these cities comprised the treatment group. Fifty-one cities were picked as the control cities (see Additional file 1: Table S1), and the births given by migrant women in these cities comprised the control group. The control cities were chosen based on several criteria: (a) being located in the same province as a treatment city; (b) having a similar level of gross domestic product (GDP) as the treatment city in the same province; and (c) having a similar number of births as the treatment city in the same province. In a robustness check, 83 control cities were chosen using propensity score matching on a host of city-level variables such as population size, per capita GDP, average wage, number of elementary schools, number of hospital beds, and average housing price.

Control variables were drawn from the CMDS, the 2010 Population Census of China, and statistical yearbooks. The individual-level control variables include maternal age, rural–urban origin of migration, intra- or interprovincial migration, duration of migration, employment status, health insurance coverage in hometown and migration destination, and whether participating mothers were raising a child in the local community before having another child. The household-level control variables included family size, intergenerational coresidence, and per capita family income. Although the EBPHS migrant pilot program was implemented at the city level, the CMDS data allowed us to identify respondents at the county level, which was a finer geographic scale. The county-level control variables included population size, per capita GDP, number of hospital beds, and number of intra- and interprovincial migrants in 2010.

### Statistical analysis

Several linear probability regression models were fitted to the dichotomous dependent variable of migrant childbirth location. For *i*th birth in county *j* and year *t*, the baseline model is specified as the following:

1 $$Y_{ijt}=\beta_0+{\textstyle\sum_{t=2010}^{2012}}\beta_t^{pre}\left(D_j\times t\right)+{\textstyle\sum_{t=2014}^{2016}}\beta_t^{post}\left(D_j\times t\right)+\theta W_{ijt}+\mu_j+\gamma_t+\epsilon_{ijt},$$

where ${D}_{j}=1$ for the treatment county and ${D}_{j}=0$ for the control county; ${W}_{ijt}$ represents all the individual-, family-, and county-level independent variables; ${\mu }_{j}$ denotes county fixed effects; ${\gamma }_{t}$ represents time fixed effects; and ${\epsilon }_{ijt}$ is the random error. Treating 2013, the year prior to the implementation of the EBPHS program, as the reference, $\sum _{t=2010}^{2012}{\beta }_{t}^{pre}$ represents the year-specific coefficients for the difference between the treatment and control groups during the pre-treatment period, while $\sum _{t=2014}^{2016}{\beta }_{t}^{post}$ represents the year-specific (and thus dynamic) treatment effects during the post-treatment period. Our Hypothesis 1 predicts the coefficients $\sum _{t=2010}^{2012}{\beta }_{t}^{pre}$ to be not statistically significant and the coefficients $\sum _{t=2014}^{2016}{\beta }_{t}^{post}$ to be statistically significant. Our Hypothesis 2 predicts that the significance level of ${\beta }_{t}^{post}$ would diminish as $t$ increases.

The second model further controlled for the interaction between time and city fixed effects as the following:

2 $$Y_{ijmt}=\beta_0+{\textstyle\sum_{t=2010}^{2012}}\beta_t^{pre}\left(D_{jm}\times t\right)+{\textstyle\sum_{t=2014}^{2016}}\beta_t^{post}\left(D_{jm}\times t\right)+\theta W_{ijmt}+\mu_j+\gamma_t+\mu_m\times\gamma_t+\epsilon_{ijmt},$$

where ${\mu }_{m}$ denotes the fixed effects at the city level (within which counties are nested), and ${\mu }_{m}\times {\gamma }_{t}$ represents the interaction between city fixed effects and time fixed effects. This interaction captures any unobserved time-varying heterogeneity between cities where the treatment and control groups are located. Note that we identified the treatment status of observations at the county level (${D}_{jm}$), and thus adding the interaction between county fixed effects and time fixed effects would absorb the treatment effects $({D}_{jm}\times t$).

The third model added the interaction between time and county-level migrant population size from the 2010 census, denoted by ${M}_{j}^{2010}$, as the following:

3 $$Y_{ijtm}=\beta_0+{\textstyle\sum_{t=2010}^{2012}}\beta_t^{pre}\left(D_{jm}\times t\right)+{\textstyle\sum_{t=2014}^{2016}}\beta_t^{post}\left(D_{jm}\times t\right)+\theta W_{ijmt}+\mu_j+\gamma_t+\mu_m\times\gamma_t+M_j^{2010}\times\gamma_t+\epsilon_{ijmt},$$

The migrant population size in 2010 was measured prior to the launch of the EBPHS program, and popular migration destinations might have been more likely to be chosen to launch the EBPHS migrant pilot program [32, 33]. Therefore, this interaction could capture potential selection bias during the pre-treatment period and ensure conditional independence of selection into the treatment versus control groups. Robust standard errors were calculated to adjust for sample clustering at the city level in all models.

## Results

### Descriptive statistics

**Fig. 1:**
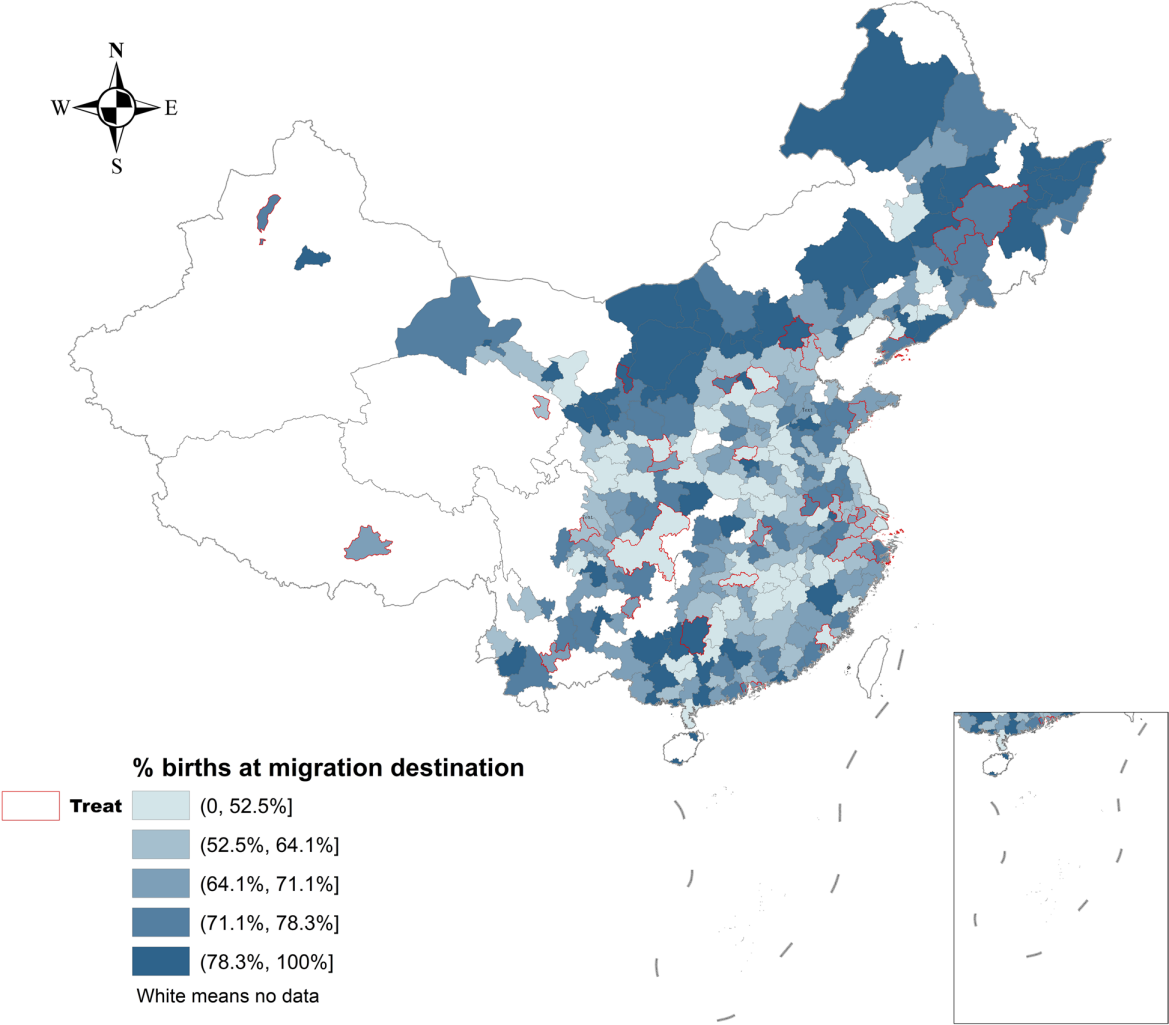
Geographic distribution of migrant childbirths at destination cities from the 2010–2016 China Migrants Dynamic Survey

Figure 1 visualizes the geographic distribution of the rate of migrant childbirths at their destination cities aggregated from the 2010–2016 CMDS data (see Additional file 1: Figure S1 for the distribution of the number of migrant childbirths). Darker colors indicate higher rates of migrant childbirths at destination cities. The areas with boundaries in red are the 40 treatment cities. The main result from Fig. 1 is that most treatment cities did not have the highest rates of migrant childbirths. This visual finding is confirmed in the descriptive statistics for the 2010–2016 pooled data, as shown in Table 1. On average, the rate of migrant childbirths at destinations was 0.67 in the treatment group, which was slightly lower than in the control group by manual matching (0.69) or propensity score matching (0.71). These descriptive results may reflect the enduring barriers for migrants to access local health care services. However, without a formal assessment, such an aggregated, descriptive pattern may obscure any significant progress made under the EBPHS migrant pilot program.

**Table 1:** Descriptive statistics of the dependent and independent variables

		Control Group﻿			
Variable	Treatment Group	By Manual Matching	*p*-value from t-test	By Propensity Score Matching	*p*-value from t-test
*Individual or family level*					
Migrant childbirth at destination (1 = *yes*; 0 = *no*)	0.698	0.713	0.022	0.716	0.120
Intergenerational coresidence (1 = *yes*; 0 = *no*)	0.095	0.098	0.479	0.098	0.226
Maternal age (year)	27.44	27.29	0.015	27.32	0.028
Parents’ highest years of education	11.73	11.24	0.000	11.24	0.000
Family size	3.479	3.540	0.000	3.550	0.000
Annual per capita family income (yuan)	2326	2147	0.000	2152	0.000
Rural origin (1 = *yes*; 0 = *no*)	0.799	0.840	0.000	0.835	0.000
Migration to urban areas (1 = *yes*; 0 = *no*)	0.74	0.629	0.000	0.611	0.000
Interprovincial migrant (1 = *yes*; 0 = *no*)	0.563	0.621	0.000	0.644	0.000
Raising a child at destination (1 = *yes*; 0 = *no*)	0.292	0.331	0.000	0.336	0.000
Duration of migration (year)	3.043	3.036	0.885	3.105	0.155
Births (*n*)	13,942	8,614		7,477	
*County level*					
Population size (10,000 persons)	436.7	397.1	0.003	403.9	0.049
GDP per capita (yuan)	84,565	77,268	0.000	75,528	0.010
Number of hospital beds	19,662	10,035	0.000	7,959	0.000
Intraprovincial migrant population in 2010	107,279	69,804	0.000	77,259	0.000
County-year observations (*n*)	1,339	975		819	

Turning to the independent variables, several descriptive findings are worth noting. First, the migrant parents had on average more than 11 years of education in both treatment and control groups, indicating selective migration by human capital. Second, the treatment group was relatively better off in terms of family background. For example, the average annual per capita family income was 2,326 yuan in the treatment group, significantly higher than in the control group by manual matching (2,147 yuan) or propensity score matching (2,152 yuan). The treatment group was also more likely to be urban-to-urban migrants rather than rural-to-urban or rural-to-rural migrants, compared with the control group. Third, on average, the counties in the treatment cities had considerably larger total and migrant populations, higher GDP per capita, and greater health care resources in terms of hospital beds than those in the control cities, regardless of the matching methods. These differences suggest that the treatment cities were carefully selected by the Chinese government to launch the EBPHS migrant pilot program because they were popular migration destinations and better equipped with economic and health care resources.

### Regression estimates

Table 2 reports the DID estimates of the causal effect of the EBPHS migrant pilot program from three regression models as described in the statistical analysis section. To maximize the statistical power, we allowed sample size to vary in different models due to missing data from additional control variables. The sample sizes after manual matching are generally smaller than those after propensity score matching because of stricter matching criterion in the former.

Across all the models in Table 2, the interaction between the indicator of the treatment group (versus the control group) and indicator of the post-treatment period (versus the pre-treatment period) represents the treatment effect of the RBPHS program on the dependent variable of migrant childbirth delivered at migration destination. When the control cities were manually selected to match the treatment cities on their background characteristics, Model 1 shows that the EBPHS program significantly increased the probability of a migrant childbirth at migration destination (as opposed to home origin) by 4.5 percentage points, net of individual- and family-level covariates, time fixed effects, and county fixed effects.

After adding the interaction between time and city fixed effects in Model 2, the coefficient of the DID estimate nearly doubled, showing that the EBPHS program significantly increased the probability of a migrant childbirth at migration destination by 8.1 percentage points. The DID estimate was roughly the same after further controlling for the interaction between time and migration size in Model 3.

**Table 2:** Differences-in-difference regression estimates

	Manual Matching						Propensity Score Matching					
	Model 1		Model 2		Model 3		Model 1		Model 2		Model 3	
	β	*p*	β	*p*	β	*p*	β	*p*	β	*p*	β	*p*
Treated × post-treatment	0.045	0.013	0.081	0.016	0.080	0.019	0.042	0.013	0.056	0.013	0.050	0.024
	(0.010, 0.081)		(0.016, 0.147)		(0.014, 0.147)		(0.009, 0.075)		(0.012, 0.100)		(0.007, 0.094)	
Individual and family controls	Yes		Yes		Yes		Yes		Yes		Yes	
Year fixed effects	Yes		Yes		Yes		Yes		Yes		Yes	
County fixed effects	Yes		Yes		Yes		Yes		Yes		Yes	
Year × city fixed effects	No		Yes		Yes		No		Yes		Yes	
Year × migration size	No		No		Yes		No		No		Yes	
Observations	22,775		22,760		22,556		24,597		24,549		24,340	

*Note*: 95% confidence intervals are within parentheses

The regression estimates remained qualitatively unchanged when control cities were selected and matched to the treatment cities based on propensity scores. Specifically, the DID estimates were statistically significant and positive across Models 1–3, although the point estimates changed slightly. For example, in the full model (i.e., Model 3), the DID estimate based on propensity score matching showed that the EBPHS program significantly increased the probability of a migrant childbirth at migration destination by only 5 percentage points, smaller than the estimate based on manual matching (8 percentage points).

**Fig. 2:**
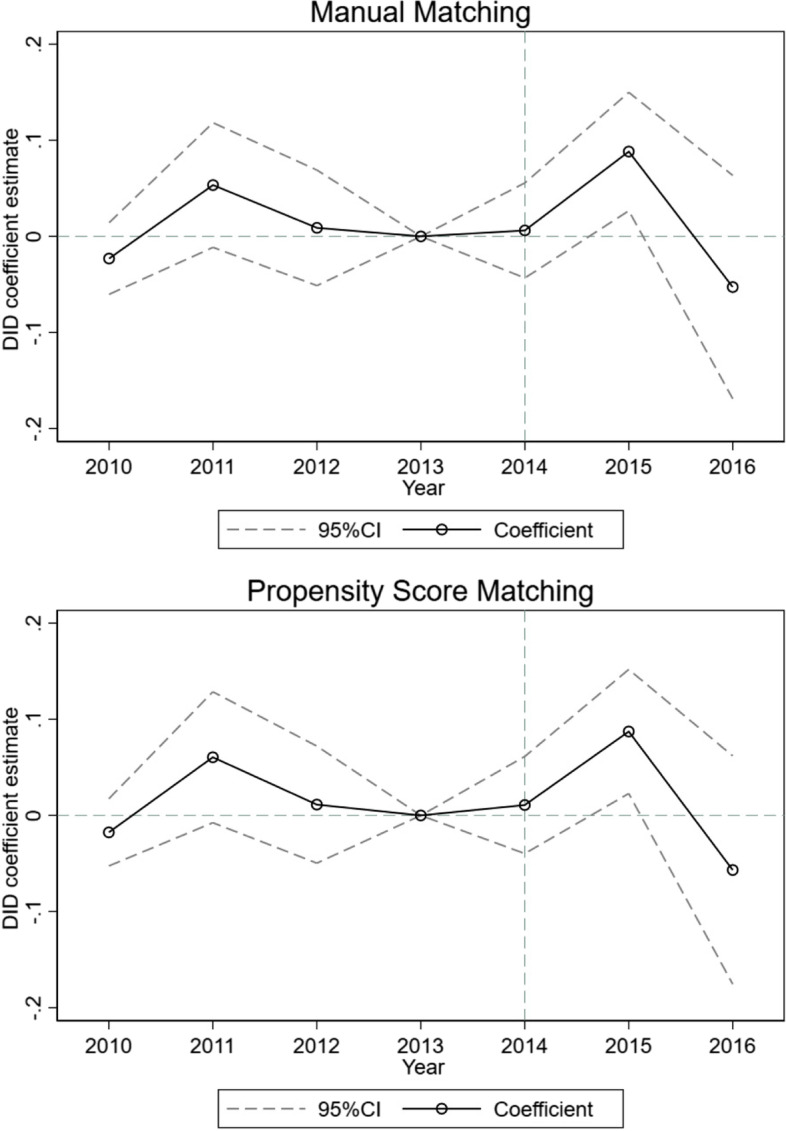
Difference-in-differences regression estimates over time with 95% confidence intervals

The DID estimates reported in Table 2 reflect the average effect of the EBPHS program during the study period of 2010–2016. To uncover the dynamic, yearly trend of the program’s impact, we reestimated Model 3 by interacting yearly dummy variables, instead of the pre- and post-treatment dummy variables, with treatment status to obtain the DID estimates. Figure 2 plots the DID coefficient estimates and the 95% confidence intervals using manual matching (top panel) and propensity score matching (bottom panel), using year 2013 as the reference. Three findings stand out. First, consistent with *Hypothesis* *1*, no DID estimate was statistically significant in the pretreatment period (2010–2012) or the year when the EBPHS migrant pilot program began (2014). Second, DID estimates were positive and statistically significant, regardless of the matching methods, for 2015, one year after the EBPHS migrant pilot program was launched, again lending support to *Hypothesis* *1*. In fact, manual matching and propensity score matching yielded similar point estimates, indicating that the pilot program increased the probability of a migrant childbirth at migration destination by nearly 9 percentage points in 2015. Third, the DID estimates were no longer significant in 2016, supporting *Hypothesis* *2* . In fact, the point estimates were negative in 2016. Together, these findings suggest that the EBPHS migrant pilot program had a short-term positive impact on improving the likelihood of migrant women giving birth at their migration destinations.

It is worth noting that the coefficient estimates for the year 2011 were positive though not statistically significant. This might be related to a national guideline released by the National Health Commission in October 2010. This guideline proposed several goals and principles for the EBPHS migrant pilot program announced three years late in December 2013.^2^ This national guideline may have prompted certain migration destination cities (a notable example being Harbin, the provincial capital city of Heilongjiang^3^) to take some early initiatives in caring for local migrant populations.

**Fig. 3:**
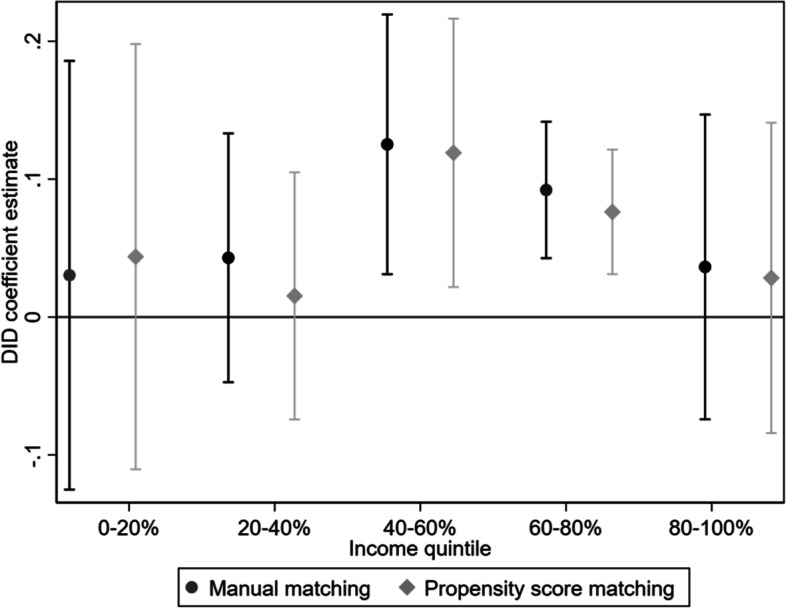
Difference-in-differences regression estimates by income quintiles with 95% confidence intervals

Last, we explored heterogeneity in the program’s effect by family income because in China, domestic migrants’ health-seeking behaviors are often constrained by the amount of economic resources at their disposal and the cost of health services at the destination city [34]. We divided the per capita family income into quintiles and estimated the program effect for each quintile. The resulting DID estimates are shown in Fig. 3. Interestingly, neither the lowest-income participants nor the highest-income participants benefit significantly from the EBPHS migrant pilot program. Those in the middle- and upper-middle-income quintiles had an increased likelihood of giving birth at migration destination through the pilot program. Specifically, the EBPHS pilot program was associated with an increased probability of childbirth at migration destination by about 9.2 to 12.5 percentage points for those in the 40–60 percentiles of family income, and about 7.6–11.9 percentage points for those in the 60–80 percentiles of family income, depending on the matching method.

### Sensitivity analysis

The DID estimates reported in Table 2 and Fig. 3 rely on the assumption of parallel trend between the treatment and control groups during the pre-treatment period. As shown in Additional file 1: Figure S2, the trends of average birth rates at migration destinations seem to converge between the treatment and control groups during the pre-treatment period of 2010–2012. However, these trends do not adjust for other control variables in our regression models. In addition, our dynamic year-specific DID estimates as shown in Fig. 2 rely on a weak assumption: no significant difference between the treatment and control groups at any time points during the pre-treatment period after adjusting for the control variables. As shown in the first column of Additional file 1: Table S2, none of the interactions between the dichotomous treatment indicator and year is statistically significant during the pre-treatment period of 2010–2012. The second column of Additional file 1: Table S2 shows that when 2014 is used as the reference year, none of the interactions between the dichotomous treatment indicator and year is statistically significant during the pre-treatment period of 2010–2013. In other words, we did not find strong evidence of the assumption of parallel trends being violated.

We conducted two sensitivity analyses to address concerns about omitted variable bias. At the individual level, employment status and health insurance coverage could affect migrant women’s decision on where to seek maternal and child delivery care [29]. For example, a migrant woman who was unemployed and did not have health insurance coverage at the migration destination tended to choose to give birth at home for lower costs. Unfortunately, the CMDS only collected these data from the primary respondent in a selected household. Therefore, we restricted the sample to migrant women who were the primary respondents and reanalyzed the data. The DID estimates of the effect of the EBPHS migrant pilot program remained substantively unchanged after controlling migrant women’s employment status and health insurance coverage (see Additional file 1: Table S3).

At the aggregate level, a migrant woman’s decision on the place of childbirth may be related to her long-term intent to permanently settle in the destination city. Such an intent often depends on how difficult it is to obtain local hukou status and how expensive the local housing market is [35]. We borrowed a city-level index of local hukou registration from a recent study [35] and city-level average housing price from the proprietary CEIC China Economic Database. We matched these two new city-level variables to the CMDS data based on the locations of cities in prefectures. Adding these city-level control variables did not qualitatively alter the DID estimates of the effect of the EBPHS migrant pilot program (see Additional file 1: Table S3).

Last, we conducted a placebo test in which we pretended that the EBPHS migrant pilot program was implemented in 2013. We then recoded 2010–2012 as the pretreatment period and 2013–2016 as the post-treatment period. We reestimated the DID regression models and expected the effect of the EBPHS program to be insignificant because in reality, the program was implemented in 2014 rather than 2013. As shown in Additional file 1: Table S4, the DID estimates showed no significant effect of the program when we pretended that it was implemented in 2011, 2012, or 2013.

## Discussion

Despite the massive rural-to-urban migration during recent decades, China’s long-lasting hukou system has restricted, if not denied, millions of migrants from receiving basic health care services at their destination cities [7–11]. The EBPHS program was envisioned as a nationwide public health campaign to address the increasingly widened gaps in accessing health care services for the general Chinese population [14]. It was not until nearly 5 years after the EBPHS was launched in 2009 that a pilot program was initiated to address the migrant population’s needs for health care. This study is among the first to formally evaluate the effectiveness of the 2014 EBPHS migrant pilot program. Our program evaluation focused on migrant women’s delivery care not only because it is an important indicator of maternal health and infant health [24–27], but also because it is one of the key policies specified in the EBPHS migrant pilot program [15, 17–21]. Empirically speaking, migrant women’s delivery care is one of the few health indicators for which microdata are available to evaluate the EBPHS program.

Drawing on nationally representative, repeated cross-sectional survey data, we examined place of delivery for more than 20,000 births by domestic migrant women during 2010–2016. Our DID regression estimates suggest that during the study period, the EBPHS migrant pilot program increased the probability of a migrant childbirth occurring at the migration destination rather than the home of origin by approximately 4–8 percentage points, depending on whether additional control variables were included or how the control group were selected and matched to the treatment group. According to our regression estimates, every 1-year increase in educational attainment was associated with about a 0.9% point increase in the probability of a migrant childbirth at a migration destination (results not shown). Therefore, the EBPHS migrant pilot program is equivalent to receiving 4–8 years of education in terms of improving migrant women’s delivery care. This is quite substantial, given that the average years of education is about 10.6 among Chinese migrants [36].

After unpacking the DID estimates averaged over 2010–2016, we found that the positive impact of the EBPHS migrant pilot program appeared to be short lived. The pilot program was officially announced in November 2013 and started to roll out in March 2014. It significantly increased the probability of a migrant childbirth at the destination by nearly 9 percentage points in 2015, but the effect lost its statistical significance in 2016. In fact, the point estimates were negative, showing that the probability of a migrant childbirth at the destination was reduced by 5.4–5.7 percentage points in 2016 compared to that in 2013, the year prior to the implementation of the EBPHS migrant pilot program. The decline in the impact of the EBPHS migrant pilot program might have resulted from the expansion of the program to other cities in 2016 [37]. The potential spillover effect of the expanded program may have attenuated the policy impact for the pilot cities included in this study.

When we explored heterogeneous effects, we found that the EBPHS migrant pilot program mainly affected the middle- and upper-middle-income groups. It is possible that the benefit coverage of the pilot program was too limited to offset the cost of delivery care,  especially for the poorest migrants. On the other hand, the richest migrants might have already accumulated sufficient economic resources to afford delivery care at the destination city, especially at private hospitals where hukou-based discrimination is less a concern. This finding suggests that the EBPHS program should expand its coverage and increasingly focus on the most vulnerable migrants.

These findings should be interpreted in light of several study limitations. First, we did not have access to CMDS data collected in recent years. Therefore, we were unable to evaluate the long-term impact of the EBPHS program. Second, we could not directly assess the receipt of the EBPHS program benefit at the individual level due to the lack of measures in the CMDS data. Therefore, our statistical inference relied on the assumption that all migrants living in the treatment cities had enrolled in the program, which may not be the case. Third, we only focused on the place of delivery as the outcome variable, which did not fully capture the quality of delivery care. Future research is needed to examine other indicators of maternal, prenatal, and postnatal care to provide a more comprehensive program evaluation.

Despite these limitations, our findings suggest that the Chinese government is capable of implementing a public health policy that is effective in providing basic delivery care to domestic migrant women. This is a significant step toward serving the long underserved migrant population who have been treated as second-class citizens by the Chinese government [38]. The preliminary evidence of the success of the EBPHS pilot program calls for its wide expansion to other parts of the country beyond the pilot cities. Furthermore, delivery care is merely one aspect of various maternal and perinatal health services targeted at migrant women and newborns. According to the latest data from China’s 2020 census, the total number of domestic migrants has increased to about 376 million (or 26.6% of the total population), nearly 70% growth from 2010, whereas the total population has only increased by 5.38% during the same period [1]. The Chinese government is now facing the challenge of expanding the variety and benefits of health care services to a broader population of migrants (e.g., annual physical examinations for migrant children and adults and routine care for older migrants with chronic conditions).

One limitation of the EBPHS program lies in its targeted population restricted to domestic migrants who are Chinese citizens. Therefore, the growing population of immigrants from other countries to China are not covered by the EBPHS program. Many foreign immigrants, especially those from African countries, have faced various barriers, stigma and even discrimination in seeking healthcare services in Chinese megacities such as Beijing and Guangzhou [12, 13, 39]. Qualitative studies have suggested certain barriers faced by foreign immigrant mothers such as discriminatory migration policies and difficulty in securing birth certification [12, 13] are similar in nature to those faced by Chinese domestic immigrants. In theory, foreign immigrants may enjoy the same benefits covered by the EBPHS program once they obtain a Chinese citizenship. However, it is unclear how many foreign immigrants are interested in becoming naturalized Chinese citizens, given that China does not recognize dual citizenships and the process of naturalization can be difficult to navigate. However, the success of the EBPHS program suggests that a similar program can be crafted to ensure better access to basic healthcare services to foreign immigrants who after all are a small but integral part of China’s public health targets.

## Conclusion

The migrant pilot program represents the first public health policy initiative by the Chinese government to address the massive migrant population’s inadequate access to basic health care services. It has made a positive impact on the delivery care for migrant women and needs to be expanded in its geographic and benefit coverages.

## Supplementary Information

**Additional file 1:** **Table S1.** List of treatment cities and control cities. **Table S2**. Tests of the differences-in-difference parallel trends. **Table S3.** Differences-in-difference regression estimates from robust check. **Table S4.** Differences-in-difference regression estimates from placebo tests. **Figure S1.** Geographic distribution of number of births in destination cities per year from the 2010–2016 China Migrants Dynamic Survey. **Figure S2.** Trends of average birth rates at migration destination.

## Data Availability

The data used for this study can be requested from: https://chinaldrk.org.cn/wjw/#/home.

## Abbreviations

CMDS: China Migrants Dynamic Survey
DID: difference-in-differences
EBPHS: Equalization of Basic Public Health Services
GDP: gross domestic product

## Footnotes

1 http://sft.fujian.gov.cn/zwgk/zxwj/sfwj/201810/t20181004_4528266.htm. https://www.whcdc.org/view/576.html. https://www.huarong.gov.cn/33159/37006/37008/37039/37279/content_1403928.html. https://wsjkw.sh.gov.cn/zhghjh/20180815/0012-57168.html.
http://cnews.chinadaily.com.cn/2015-04/22/content_20509229.htm.

2
http://www.nhc.gov.cn/zwgk/wtwj/201306/f7794b5d6b9b40148d3643f87e14c5b1.shtml.

3
http://www.harbin.gov.cn/art/2011/12/10/art_4489_60667.html.
