# Supplementary material for: Does China’s Equalization of Basic Public Health Services policy improve delivery care for migrant women?
Source: BMC Public Health. 2023 Jan 11;23:74. doi: 10.1186/s12889-022-14950-8 (PMC9832805; doi:10.1186/s12889-022-14950-8)

Additional file 1

Table S1. List of treatment cities and control cities.

|  |  | Control City | |
| --- | --- | --- | --- |
| Province | Treatment City | By Manual Matching | By Propensity Score Matching |
| Beijing | Chaoyang, Fengtai | Haidian, Daxing | Haidian, Daxing |
| Tianjin | Binhai new area, Jinnan | Dongli, Beichen | Dongli, Beichen |
| Shanghai | Minhang, Yangpu, Songjiang, Baoshan | Qingpu, Fengxian, Jiading, Pudong new area | Qingpu, Fengxian, Jiading, Pudong new area |
| Chongqing | Yubei | Jiulongpo | Jiulongpo |
| Hebei | Shijiazhuang | Langfang, Tangshan | Langfang, Tangshan, Baoding, Cangzhou, Qinhuangdao, Handan |
| Shanxi | Taiyuan | Shuozhou, Jincheng | Jincheng, Linfen, Datong, Xinzhou, Jinzhong, Changzhi, Yangquan, Yuncheng |
| Liaoning | Dalian | Panjin | Liaoyang, Anshan |
| Jilin | Changchun | Songyuan | Songyuan, Siping |
| Heilongjiang | Harbin | Daqing, Mudanjiang | Daqing |
| Jiangsu | Nanjing, Suzhou, Wuxi | Changzhou, Nantong, Yangzhou, Zhenjiang | Changzhou, Nantong, Yangzhou, Zhenjiang, Xuzhou, Yancheng |
| Zhejiang | Hangzhou, Ningbo, Jiaxing, Shaoxing | Wenzhou, Jinhua, Taizhou, Huzhou, Zhoushan | Wenzhou, Jinhua, Taizhou, Huzhou, Zhoushan, Lishui, Quzhou |
| Anhui | Hefei | Wuhu | Wuhu, Huaibei, Huainan, Maanshan |
| Fujian | Xiamen Quanzhou | Fuzhou, Longyan, Sanming | Fuzhou, Longyan, Zhangzhou |
| Shandong | Qingdao | Weihai | Weihai, Dongying, Linyi, Dezhou, Taian, Jining , Zibo, Binzhou, Weifang, Yantai, Liaocheng |
| Henan | Zhengzhou | Luoyang, Sanmenxia | Luoyang, Pingdingshan |
| Hunan | Zhangsha | Zhuzhou, Xiangtan | Zhuzhou, Huaihua, Hengyang, Chenzhou |
| Guangdong | Shenzhen Zhongshan | Guangzhou, Foshan, Dongguan | Foshan, Dongguan, Huizhou, Jiangmen, Zhanjiang, Zhuhai, Maoming |
| Guangxi | Guilin | Liuzhou | Liuzhou |
| Sichuan | Chengdu | Deyang, Mianyang | Deyang, Mianyang, Yibin, Panzhihua |
| Guizhou | Guiyang | Zunyi, Liupanshui | Zunyi |
| Yunnan | Yuxi | Kunming | Kunming, Qujing |
| Shaanxi | Xi’An, Xianyang | Yulin, Yanan | Yulin, Yanan, Baoji, Hanzhong |
| Xinjiang | Karamay | Urumchi | Urumchi |
| Hubei | Wuhan | Xiangyang, Yichang |  |
| Qinghai | Xining | Haidong |  |
| Ningxia | Yinchuan | Shizuishan |  |
| Tibet | Lhasa |  |  |
| Inner Mongolia |  |  | Hohhot, Chifeng, Tongliao |
| Jiangxi |  |  | Shangrao, Jiujiang, Nanchang, Ganzhou |
| Hainan |  | Haikou |  |

Table S2. Tests of the differences-in-difference parallel trends.

|  | Dependent Variable: Migrant childbirth at destination  (1 = *yes*; 0 = *no*) | | | |
| --- | --- | --- | --- | --- |
| VARIABLES | Using 2013 as the reference | | Using 2014 as the reference | |
|  | β | *p* | β | *p* |
| $D_{j}\times T_{t}^{2010}$ | -0.023 | 0.221 | -0.023 | 0.229 |
|  | (-0.060, 0.014) | | (-0.061, 0.015) | |
| $D_{j}\times T_{t}^{2011}$ | 0.053 | 0.105 | 0.053 | 0.104 |
|  | (-0.011, 0.118) | | (-0.011, 0.118) | |
| $D_{j}\times T_{t}^{2012}$ | 0.009 | 0.769 | 0.009 | 0.774 |
|  | (-0.051, 0.069) | | (-0.053, 0.071) | |
| $D_{j}\times T_{t}^{2013}$ |  |  | 0.008 | 0.777 |
|  |  |  | (-0.049, 0.066) | |
| $D_{j}\times T_{t}^{2014}$ | 0.006 | 0.805 |  |  |
|  | (-0.043, 0.056) | |  | |
| $D_{j}\times T_{t}^{2015}$ | 0.088** | 0.006 | 0.088** | 0.005 |
|  | (0.027, 0.150) | | (0.028, 0.149) | |
| $D_{j}\times T_{t}^{2016}$ | -0.053 | 0.368 | -0.053 | 0.365 |
|  | (-0.169, 0.063) | | (-0.168, 0.063) | |
| Constant | 0.244 | 0.227 | 0.246 | 0.239 |
|  | (-0.156, 0.645) | | (-0.166, 0.658) | |
| R-squared | 0.118 |  | 0.118 |  |
| Individual and family controls | YES |  | YES |  |
| Year fixed effects | YES |  | YES |  |
| County fixed effects | YES |  | YES |  |
| Year × city fixed effects | YES |  | YES |  |
| Year × migration size | YES |  | YES |  |
| Observations | 22,595 | | 22,595 | |

*Note*: 95% confidence intervals are within parentheses.

Table S3. Differences-in-difference regression estimates from robust check.

|  | Manual Matching | | | | Propensity Score Matching | | | | |
| --- | --- | --- | --- | --- | --- | --- | --- | --- | --- |
|  | Subsample | | Full Sample | | Subsample | | Full Sample | |  |
|  | β | *p* | β | *p* | β | *p* | β | *p* |  |
| Treated × post-treatment | 0.085 | 0.008 | 0.058 | 0.0001 | 0.067 | 0.016 | 0.039 | 0.005 |  |
|  | (0.023, 0.148) | | (0.028, 0.089) | | (0.013, 0.122) | | (0.012, 0.067) | |  |
| Women’s employment status: work = 1 | 0.060 | 0.0001 |  |  | 0.064 | 0.0001 |  |  |  |
|  | (0.038, 0.082) | |  | | (0.043, 0.085) | |  |  |  |
| Women Owned medical insurance in hometown | -0.025 | 0.018 |  |  | -0.032 | 0.002 |  |  |  |
|  | (-0.045, -0.004) | |  |  | (-0.052, -0.013) | |  |  |  |
| Women Owned medical insurance in destination | -0.035 | 0.016 |  |  | -0.029 | 0.025 |  |  |  |
|  | (-0.063, -0.007) | |  |  | (-0.055, -0.004) | |  |  |  |
| City-level hukou index |  |  | 0.087 | 0.058 |  |  | 0.078 | 0.070 |  |
|  |  |  | (-0.003, 0.178) | |  |  | (-0.006, 0.161) | |  |
| City-level average housing price |  |  | 0.009 | 0.875 |  |  | 0.049 | 0.477 |  |
|  |  |  | (-0.109, 0.127) | |  |  | (-0.088, 0.186) | |  |
| Time (year) fixed effects | Yes |  | Yes |  | Yes |  | Yes |  |  |
| County fixed effects | Yes |  | Yes |  | Yes |  | Yes |  |  |
| Time × city fixed effects | Yes |  | Yes |  | Yes |  | Yes |  |  |
| Time × migration size | Yes |  | Yes |  | Yes |  | Yes |  |  |
| Observations | 11,730 | | 12,566 | | 15,548 | | 16,175 | |  |

*Note*: 95% confidence intervals are within parentheses.

Table S4. Differences-in-difference regression estimates from placebo tests.

|  | Manual Matching | | | | | | Propensity Score Matching | | | | | | |
| --- | --- | --- | --- | --- | --- | --- | --- | --- | --- | --- | --- | --- | --- |
|  | Treatment in 2011 | | Treatment in 2012 | | Treatment in 2013 | | Treatment in 2011 | | Treatment in 2012 | | Treatment in 2013 | |  |
|  | β | *p* | β | *p* | β | *p* | β | *p* | β | *p* | β | *p* |  |
| Treated × post-treatment | 0.027 | 0.265 | 0.037 | 0.142 | 0.017 | 0.494 | 0.004 | 0.895 | 0.021 | 0.450 | 0.002 | 0.947 |  |
|  | (-0.021 - 0.075) | | (-0.013 - 0.086) | | (-0.033 - 0.067) | | (-0.051 - 0.059) | | (-0.033 - 0.075) | | (-0.051 - 0.055) | |  |
| Individual and family controls | Yes |  | Yes |  | Yes |  | Yes |  | Yes |  | Yes |  |  |
| Time (year) fixed effects | Yes |  | Yes |  | Yes |  | Yes |  | Yes |  | Yes |  |  |
| County fixed effects | Yes |  | Yes |  | Yes |  | Yes |  | Yes |  | Yes |  |  |
| Time × city fixed effects | Yes |  | Yes |  | Yes |  | Yes |  | Yes |  | Yes |  |  |
| Time × migration size | Yes |  | Yes |  | Yes |  | Yes |  | Yes |  | Yes |  |  |
| Observations | 23,508 |  | 23,508 |  | 23,508 |  | 25,343 |  | 25,343 |  | 25,343 |  |  |

*Note*: 95% confidence intervals are within parentheses.

Figure S1. Geographic distribution of number of births in destination cities per year from the 2010–2016 China Migrants Dynamic Survey.


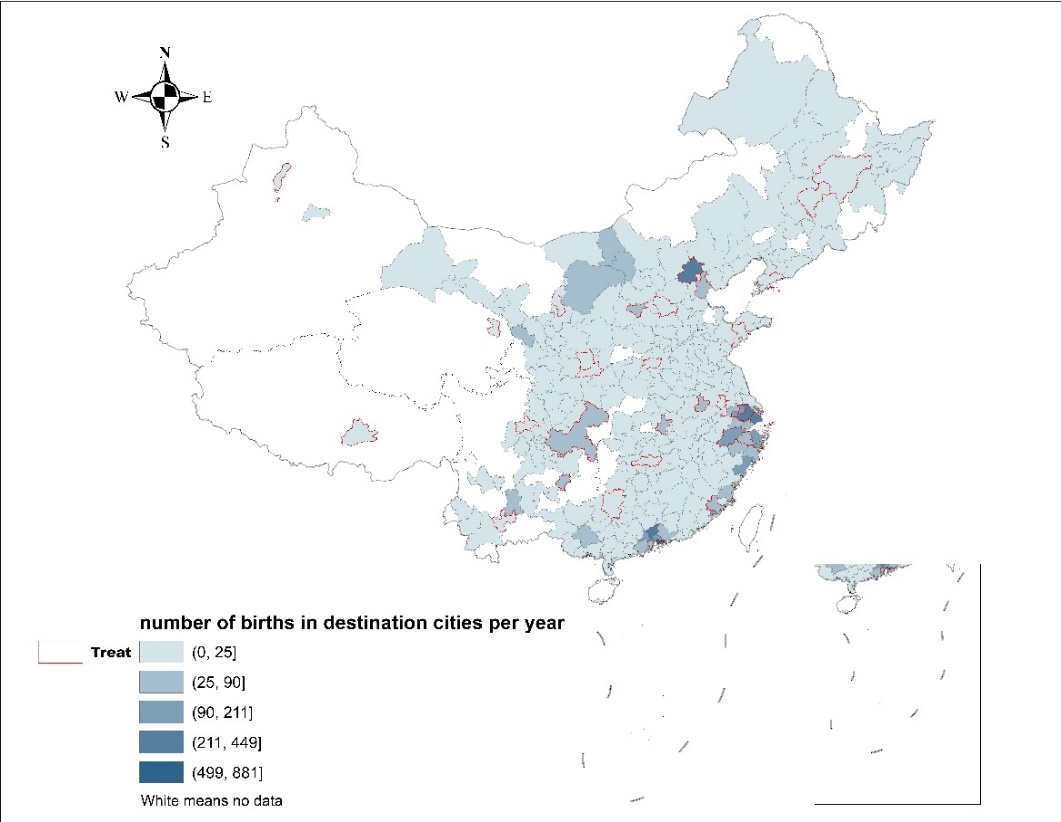


Figure S2. Trends of average birth rates at migration destination.


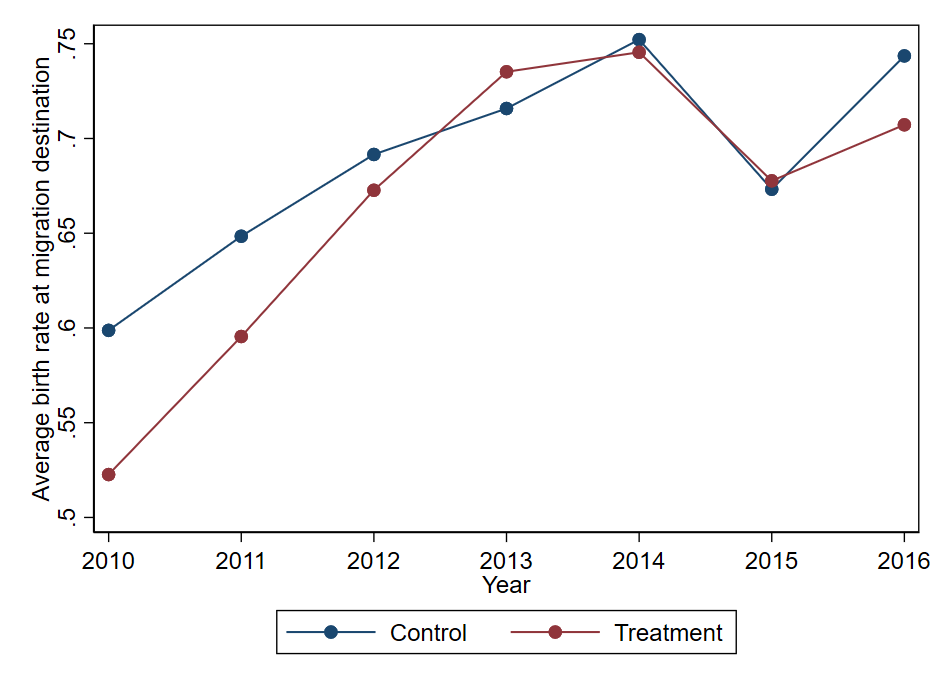

Supplement: Supplementary file 1 — Additional file 1: Table S1. List of treatment cities and control cities. Table S2. Tests of the differences-in-difference parallel trends. Table S3. Differences-in-difference regression estimates from robust check. Table S4. Differences-in-difference regression estimates from placebo tests. Figure S1. Geographic distribution of number of births in destination cities per year from the 2010–2016 China Migrants Dynamic Survey. Figure S2. Trends of average birth rates at migration destination. [file 12889_2022_14950_MOESM1_ESM.docx]
